# Solid-State Preparation of Metal and Metal Oxides Nanostructures and Their Application in Environmental Remediation

**DOI:** 10.3390/ijms23031093

**Published:** 2022-01-20

**Authors:** Carlos Diaz, Maria Luisa Valenzuela, Miguel Á. Laguna-Bercero

**Affiliations:** 1 Departamento de Química, Facultad de Ciencias, Universidad de Chile, Las Palmeras 3425, Ñuñoa, Casilla 653, Santiago 7800003, Chile; 2 Instituto de Ciencias Químicas Aplicadas, Grupo de Investigación en Energía y Procesos Sustentables, Facultad de Ingeniería, Universidad Autónoma de Chile, Av. El Llano Subercaseaux 2801, Santiago 8900000, Chile; maria.valenzuela@uautonoma.cl; 3 Instituto de Nanociencia y Materiales de Aragón (INMA), CSIC-Universidad de Zaragoza C/Pedro Cerbuna 12, 50009 Zaragoza, Spain; malaguna@unizar.es

**Keywords:** metal oxides, nanostructures, solid state, photocatalyst, ambient remediation

## Abstract

Nanomaterials have attracted much attention over the last decades due to their very different properties compared to those of bulk equivalents, such as a large surface-to-volume ratio, the size-dependent optical, physical, and magnetic properties. A number of solution fabrication methods have been developed for the synthesis of metal and metal oxides nanoparticles, but few solid-state methods have been reported. The application of nanostructured materials to electronic solid-state devices or to high-temperature technology requires, however, adequate solid-state methods for obtaining nanostructured materials. In this review, we discuss some of the main current methods of obtaining nanomaterials in solid state, and also we summarize the obtaining of nanomaterials using a new general method in solid state. This new solid-state method to prepare metals and metallic oxides nanostructures start with the preparation of the macromolecular complexes chitosan·Xn and PS-co-4-PVP·MXn as precursors (X = anion accompanying the cationic metal, n = is the subscript, which indicates the number of anions in the formula of the metal salt and PS-co-4-PVP = poly(styrene-co-4-vinylpyridine)). Then, the solid-state pyrolysis under air and at 800 °C affords nanoparticles of M°, M_x_O_y_ depending on the nature of the metal. Metallic nanoparticles are obtained for noble metals such as Au, while the respective metal oxide is obtained for transition, representative, and lanthanide metals. Size and morphology depend on the nature of the polymer as well as on the spacing of the metals within the polymeric chain. Noticeably in the case of TiO_2_, anatase or rutile phases can be tuned by the nature of the Ti salts coordinated in the macromolecular polymer. A mechanism for the formation of nanoparticles is outlined on the basis of TG/DSC data. Some applications such as photocatalytic degradation of methylene by different metal oxides obtained by the presented solid-state method are also described. A brief review of the main solid-state methods to prepare nanoparticles is also outlined in the introduction. Some challenges to further development of these materials and methods are finally discussed.

## 1. Introduction

**Figure 1:**
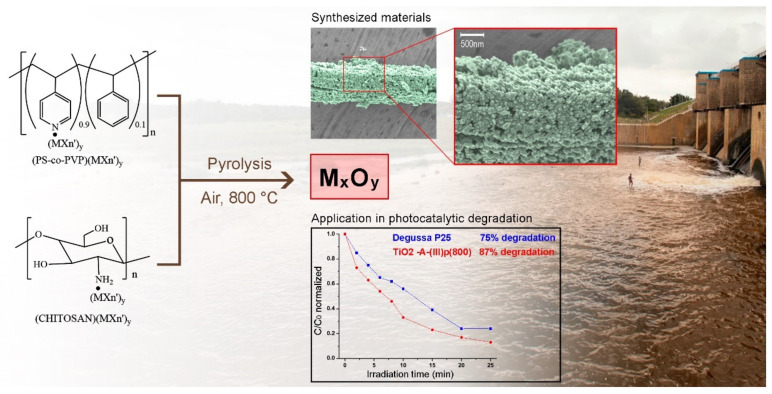
Schematic representation of the solid-state method and its application in environmental remediation.

Over the past few decades, nanoscale particles have caused much interest due to their distinct chemical, physical and biological properties. A variety of nanoparticles (NPs) with various shapes such as spheres, nanotubes, nanohorns, and nanocages, made different materials, from organic dendrimers, liposomes, gold, carbon, semiconductors, silicon to iron oxide, have already been fabricated and explored in many scientific fields, including chemistry, material sciences, physics, medicine, and electronics [1,2,3,4,5]. In this sense, a number of solution methods have been developed for the synthesis of metal and metal oxides nanoparticles [2,3], but few solid states have been reported [6]. The application of nanostructured materials to electronic solid-state devices or to high-temperature technology requires, however, adequate solid-state methods for obtaining nanostructured materials [7,8,9,10,11]. For instance, recent studies reported that the evaporation of solvent to obtain Au nanoparticles in solid state (for adequate incorporation to a solid-state device) results in 3D Au superstructures with properties different to those of Au nanostructures [12]. Thus, solvent-less synthesis of nanostructures is highly significant due to its economical, eco-friendly, and industrially viable nature. Then, the development of new solid-state methods to prepare metallic nanostructured materials is a constant challenge. We have previously informed a new solid-state method to synthesize metallic nanostructures nanomaterials from the pyrolysis of metallic and organometallic derivatives of poly and oligophosphazene under air at 800 °C [13,14,15,16,17]. Nanostructured metals (M), metal oxides (M_x_O_y_), and salts (M_x_P_y_O_z_, where P = malonates, succinates, etc.) are obtained, depending on the nature of the metal. Another method when the respective metallic or organometallic derivative is not possible to prepare uses mixtures such as MLn/[NP(O_2_C_12_H_8_)]_3_ [18,19,20]. In this case, pure phase metallic nanoparticles are obtained. However, in several of these systems, the M or M_x_O_y_ phase is accompanied by a phosphate phase. These methods have been discussed in detail in several publications [13,14,15,16,17,18,19,20,21,22,23]. In this chapter, we will discuss a novel preparation method of metallic and metal oxides nanostructured particles starting from the macromolecular chitosan·MXn and PS-co-4-PVP·MXn precursor and subsequent solid-state pyrolysis at 800 °C under air (see Figure 1), including their application in environmental remediation. In addition, we will present a brief discussion of some recent solid-state methods to prepare metallic and metal oxides nanoparticles.

In this review, we first present studies of different methods for obtaining solid-state nanomaterials collected from the literature. In addition, a summary of the results obtained in the preparation of nanomaterials using our developed solid-state method is also presented. Finally, results regarding their application in photocatalysis will be discussed for the different nanostructured metal oxides.

## 2. A General Survey of Methods for Preparation of Metal Oxide Nanoparticles

### 2.1. The Korgel’s Method

**Figure 2:**
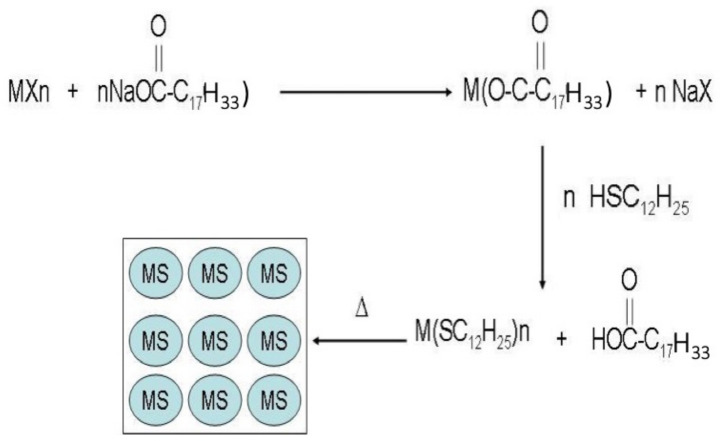
Squematic representation of Korgel’s method. The first two steps are in solution and correspond to the preparation of the solid precursor. The third step corresponds to the solid thermolysis of the M(SC_12_H_25_)_n_ precursor.

This method consists of the thermolysis of Metal-Thiolate complexes. The products are metal-sulfide nanoparticles with monodispersed and shape distributions. Briefly, the precursor is prepared by a biphasic reaction between a metallic salt and dodecanethiol using sodium octanoate as a phase transfer catalyst to solubilize the metal cationic in a water/chloroform mixture (see Figure 2). The aqueous phase is discarded, and the organic solvent is evaporated. The waxy solid is heated in the air up to 140–240 °C depending on the metal used. Then, the material is redispersed in chloroform to eliminate the rest of the dodecanethiol stabilizer. Different metal-sulfide nanoparticles such as Cu_2_S [24,25], NiS [26], or Bi_2_S_3_ [27], among others, were prepared using this method. In another approach, thermolysis of the related stearate M(OOCC_17_H_33_)_n_ affords the respective metal oxides [28].

### 2.2. The Molecular and Macromolecular Complex Decomposition Method

This method consists simply of the solid-state thermal decomposition of a molecular complex under air at temperatures about 800 °C. Several examples of decomposition of [Ni(en)_3_](NO_3_)_2_ [29], Nickel dimethylglyoximate [30], [bis(2-hydroxy-1-naphtaldehyde) manganese(II)] [31], MnOOH (in presence of Mn_3_O_4_) are used to produce NiO and Mn_3_O_4_, respectively [32]. In addition, ZnO was obtained from thermolysis of bis(acetylacetonate)Zn(II) [33] and Co_3_O_4_ from solid-state pyrolysis of the [Co(NH_3_)_6_](NO_3_)_2_ complex [34].

A most general solid-state method to prepare Mn, Fe, Co, Ni, Cu, and Zn oxides has been reported from the solid-state decomposition of the metal transition malonates MCH_2_C_2_O_4_·xH_2_O and transition succinates M(CH_2_)_2_C_2_O_4_·xH_2_O [35].

Metal polymeric materials have also been used as precursors of nanostructured metal oxides. For instance, pyrolysis of the [Ru(CO)_4_]_n_ polymer affords ruthenium nanofibers [36]. Pyrolysis of poly(ferrocenylsilanes) affords α-Fe nanoparticles with 20 ± 5 Å in size [37]. In addition, pyrolysis of poly(ferrocenylsilanes) yields interesting ferromagnetic ceramic composites at 500–1000 °C containing Fe particles in a SiC/C matrix [38].

Metal ligand coordination polymers have also been proposed as useful precursors of nanostructured metal oxides. Pyrolysis at 800 °C of a Ga-acetate polymer, {[Ga(µ-OH)(µ-O_2_CCH_3_)_2_]·HOAc·H_2_O}_n_ gives rise to nanostructured Ga_2_O_3_ [39].

**Figure 3:**
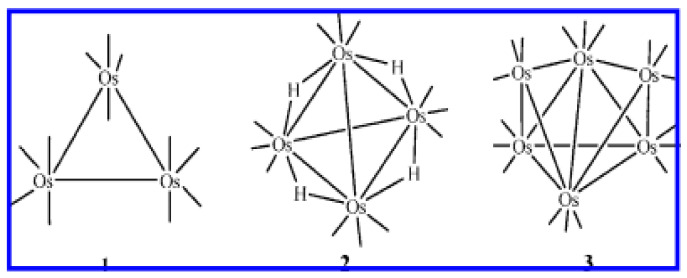
Schematic representation of the structures of Os_3_(CO)_12_ (**1**), Os4-(µ-H)_4_(CO)_12_ (**2**), and Os_6_(CO)_18_ (**3**). Adapted from reference [40].

Furthermore, pyrolysis of some miscellaneous solid precursors has been reported. The Os clusters Os_3_(CO)_12_ (**1**), Os4-(µ-H)_4_(CO)_12_ (**2**), and Os_6_(CO)_18_ (**3**), whose schematic representation of their skeletal is shown in Figure 3 [40].

Pyrolysis of these clusters affords Os nanoparticles in the 1 to 10 nm size range. A similar approach was reported using ruthenium clusters, where Ru nanoparticles were obtained.

This method was also proposed for the fabrication of different perovskites. For example, LaMnO_3±δ_ was prepared by thermal treatment of the LaMnOx/C precursors [41]. The solid-state gelation synthesis route was extended to a wide range of interesting perovskite oxides, including LaMnO_3_, LaFeO_3_, LaNiO_3_, LaCoO_3_, La_0.5_Sr_0.5_CoO_3_, and La_0.5_Sr_0.5_Co_0.5_Fe_0.5_O_3_.

Other composites, including perovskites such as CsPbBr_3_−Al_2_O_3_, were synthetized by calcination of the mixture CsBr/PbBr_2_/AIP = 1:1:30 at 800 °C for 10 min under a nitrogen atmosphere. The as-obtained perovskites possessed a high quantum yield up to 70%, narrow emission line width of 25 nm, and outstanding thermal stability [42].

In addition, perovskites of the type of nanostructured BaTiO_3_ and SrTiO_3_ have been prepared on a large scale by a solid state by reaction of the strontium or barium oxalate with TiO_2_ in anatase phase at 820 °C [43].

Supramolecular metal structures have also been used as useful precursors for nanostructured metal oxides. For instance, Na_6_[Fe_2_(µ-O)(µ-CO_3_)-(chnida)_2_]·13.5H_2_O (1; chnida = N-[(3-carboxy-2-oxy-naphthyl)methylene]iminodiacetate), after heating at 1100 °C affords nanoparticles of NaFeO_2_ [44].

Additional examples of heterostructures include Co_3_O_4_/ZnO composites prepared by thermal treatment of the Co_3_O_4_/Zn(OH)_2_ precursor. The as-prepared heterostructure exhibits a high photocatalytic activity toward Rhodamine B dye higher than ZnO [45]. In a similar way, Fe_3_O_4_@M (where M = Au, Ag, and Au-Ag alloy) core-shell nanostructures were synthesized to gram scale in the laboratory conditions by a similar high-temperature solid-state method [46]. The method consists of the thermal treatment of the solid-state mixture of the respective metallic salts with Fe_3_O_4_. The as-obtained Fe_3_O_4_@M nanocomposites exhibited catalytic activity in the obtention of H_2_ from NH_3_BH_3_ and NaBH_4_.

Thermal decomposition of organometallic precursors is another usual solid-state route to prepare nanostructured metal oxides. For instance, thermal treatment of the ferrocene carboxaldehide gives Fe_2_O_3_ nanoparticles (hematite phase) with an average size of 5 nm [47].

Another synthesis strategy arises from the solid-state thermolysis of the metal organic framework (MOFs) [48]: Zn(ADA)(4,4′-bipy)_0.5_, [Mn_2_(hfbba)_2_(3-mepy)]●(H_2_O)] [Mg_3_(O_2_CH)_6_I[NH(CH_3_)_2_]_0.5_], [Cd(ADA)(4,4′-bipy)_0.5_]●(DMF)], [Cd(tdc)(bpy)(H_2_O)]_n_ [Cu_3_(TMA)_2_(H_2_O)_3_]_n_ and [Co_6_(BTC)_2_(HCOO)_6_(DMF)_6_] affords the nanostructured of Cu/CuO, Co/Co_3_O_4_, ZnO, Mn_2_O_3_, MgO, CdS/CdO. These nanoparticles dispersed in a carbon matrix showed promising H_2_ and CO_2_ adsorption properties depending on the environment used for the thermolysis of MOFs.

In a similar manner, additional interesting precursors were the mixture of glucose-urea-transition metal, which, after pyrolysis, gave rise to a series of 2D porous metal oxides La_0.5_Sr_0.5_Co_0.8_Fe_0.2_O_3_, Co_3_O_4_, NiCo_2_O_4_, and RuO_2_ and 1D nanowire Ba_0.5_Sr_0.5_Co_0.8_Fe_0.2_O_3_, which were obtained by calcination in the air [49]. The precursors were prepared first, synthesizing the glucose-urea deep eutectic solvent on which the metallic precursors were added. The as-prepared materials exhibit high activity for the electrochemical oxygen evolution.

Finally, polynuclear clusters have also been used in the preparation of Cr_2_O_3_ nanoparticles using a solid-state method starting from direct thermal decomposition of [Cr_3_O(CH_3_CO_2_)_6_(H_2_O)_3_]NO_3_●CH_3_COOH ([Cr_3_O]) in Ar atmosphere [50]. The nanoplates embedded in carbon show an efficient enhanced electrochemical performance.

Another similar example is the solid-state preparation of CoFe_2_O_4_/C from thermal treatment of a heterometallic trinuclear [CoFe_2_O(CH_3_COO)_6_(H_2_O)_3_·2H_2_O] [51] complex, showing an average particle size of 50 nm coated with carbon on the surface.

### 2.3. A Novel Solid-State Approximation

Recently, we have proposed a novel solid-state approximation including two steps [52,53,54,55,56,57,58,59,60,61,62,63,64,65,66,67]:

1. The preparation of macromolecular precursors of general formula chitosan●MXn and PS-co-4-PVP·MXn;
2. Pyrolysis of the chitosan·MXn and PS-co-4-PVP●MXn.

The preparation of the macromolecular chitosan●MXn and PS-co-4-PVP·MXn precursors is performed by a simple coordination reaction using dichloromethane as solvent at room temperature (see Equations (1) and (2)).

(1) $$Mx_{n}+Chitosan\overset{CH_{2}Cl_{2}}{\to }Chitosan\cdot Mx_{n}$$

(2) $$Mx_{n}+PS-co-4-PVP\overset{CH_{2}Cl_{2}}{\to }PS-co-4-PVP\cdot Mx_{n}$$

Due to the large coordination site of the polymer (e.g., about 350 for the chitosan Mw = 61.000) and the insolubility of some reactants, the reaction is slow. Ensuring a high percentage of coordination takes about two weeks. However, the insoluble product is easily separable by decantation and dried in vacuum. Products are stable, solid, and with the characteristic color of the precursor metallic salt (e.g., white for Ti, green for Cr and Ni, etc.).

Pyrolysis of the solid chitosan·MXn and PS-co-4-PVP·MXn precursors under air at 800 °C, usually in a ceramic crucible, affords the nucleation of M_X_O_Y_ metal oxides. The obtained results will be shown in order of the periodic table.

#### 2.3.1. Transition Metal (First Row)

##### Titanium

Titanium dioxide (TiO_2_) is a well-known semiconductor, which can crystallize in eight different polymorphic forms [67], e.g., rutile, anatase, brookite, TiO2-B (bronze). In particular, anatase is known to be a potential solar-driven photocatalyst active for the photodegradation of various dye contaminants [68]. For this study, we selected the following precursors: (Chitosan)·(Cp_2_TiCl_2_) (I), (PS-co-4-PVP)·(Cp_2_TiCl_2_) (II), (chitosan)·(TiOSO_4_) (III) and (PS-co-4-PVP)·(TiOSO_4_) (IV) and (chitosan)·(Ti(acac)_2_) (V) and (PS-co-4-PVP)·(Ti(acac)_2_) (VI). As shown in Table 1, the obtained polymorph of the pyrolytic products depends on the nature of both the polymer and the metallic precursor. It is noteworthy that the chitosan polymer affords a pure rutile phase using Cp_2_TiCl as metallic salt joined to the polymeric chain and pure anatase using TiO^+2^ joined to the polymeric chain. The polymer PS-co-4-PVP induces a mixture of rutile and anatase phases. A summary of the different products obtained using the different precursors at several temperatures, average sizes, band gaps, and morphologies are displayed in Table 1.

Interestingly, the product from the (TiOSO4)•(Chitosan) precursor at all temperatures affords a pure anatase phase. In contrast, the solution synthesis methods almost always give phase mixtures such as anatase/rutile or anatase/brookite. As it will be later discussed in detail, the photocatalytic activity toward blue methylene was tested for TiO_2_ obtained from all precursors. The best photocatalyst, the anatase obtained from the (chitosan)•(TiOSO_4_) precursor at 800 °C, achieved a 98% discoloration rate in only 25 min when the pH of the solution was 9.5, improving the efficiency of the standard Degussa P25 photocatalyst without the addition of other phases or dopants.

##### Vanadium

**Figure 4:**
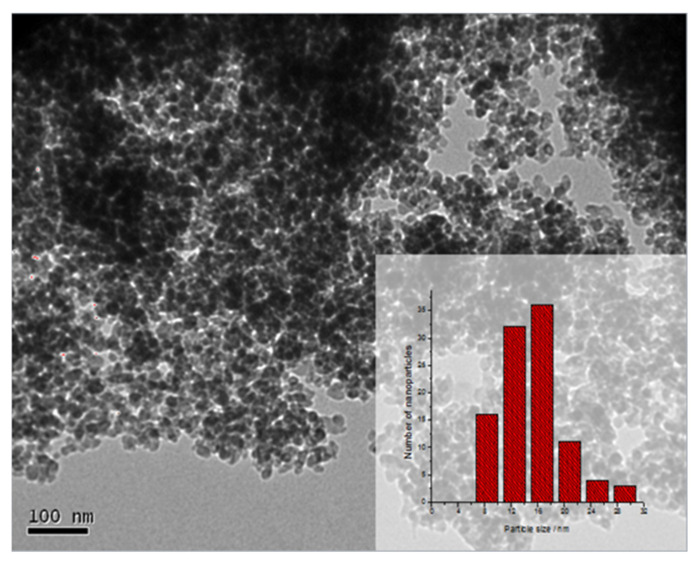
TEM image of the pyrolytic product from VCl_3_•chitosan (1:5) and their histogram (inset) adapted from reference [72].

Owing to its different oxidation states, vanadium has several metal oxides being the most common VO, V_2_O_3_, VO_2_, and V_2_O_5_ [69]. The most common morphology for V_2_O_5_ is the nucleation in lamellar form. Different morphologies as bundles with spindle-like morphologies [70], V_2_O_5_ macro-plates, nanoribbons, nanowires, or nanorods have been reported [71]. For our study, we have selected the chitosan•(VCl_3_)_n_ and PS-co-4-PVP•(VCl_3_)_n_ precursors. The same pyrolytic V_2_O_5_ product was obtained for precursors in molar ratios 1:1 and 1:5 [72]. The smallest nanoparticles were obtained for the chitosan precursor in a 1:5 ratio (see Figure 4), where nanoparticles as small as 8 nm were observed.

Although several solution methods to prepare nanoparticles of V_2_O_5_ [69,70,71] have been described, only a few solid-state methods have been reported [73,74,75]. For example, solid-state thermal decomposition of [NH_4_V_3_(OH)_6_(SO_4_)_2_] affords single-crystalline V_2_O_5_ nanoparticles, used as cathode material for lithium-ion batteries [73]. In addition, pyrolysis of the solid [N_3_P_3_(OC_6_H_5_)_5_(OC_5_H_4_N·Cp_2_VCl][PF_6_] affords mixtures of V_2_O_5_/VO(PO_3_)_2_ [74].

##### Chromium, Molybdenum, and Tungsten

The most common oxides of the VI group are Cr_2_O_3_, MoO_3_ and WO_3_. These metal oxides can be obtained using the same pyrolitic method, from the respective (chitosan)·(CrCl_3_)_x_ and PS-co-4-PVP·(CrCl_3_)_x_, PS-co-4-PVP●(MoCl_4_)_n_ and chitosan●(MoCl_4_)_n_, and from (chitosan)●WCl_4_ and PS-co-4-VP·WCl_4_ precursors [76]. For Cr_2_O_3_ nanoparticles, the size can be controlled by the metal/polymer ratio decreasing in the order 1:1 > 1:5 > 1:10.

##### Manganese

**Figure 5:**
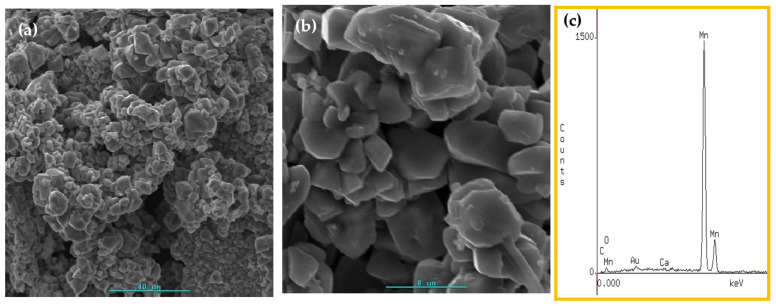
SEM image at two magnification levels (**a**,**b**) for Mn_2_O_3_ and their EDS analysis (**c**).

For this study, we selected the precursors chitosan·(MnCl_2_)_n_ and PS-co-4-PVP·(MnCl_2_)_n_. XRD analysis clearly shows the presence of Mn_2_O_3_ [77]. Figure 5 shows SEM analysis, indicating the presence of dense grains, some of them fused in 3D arrangement. EDS analysis confirmed the formation of manganese oxide.

##### Iron

**Figure 6:**
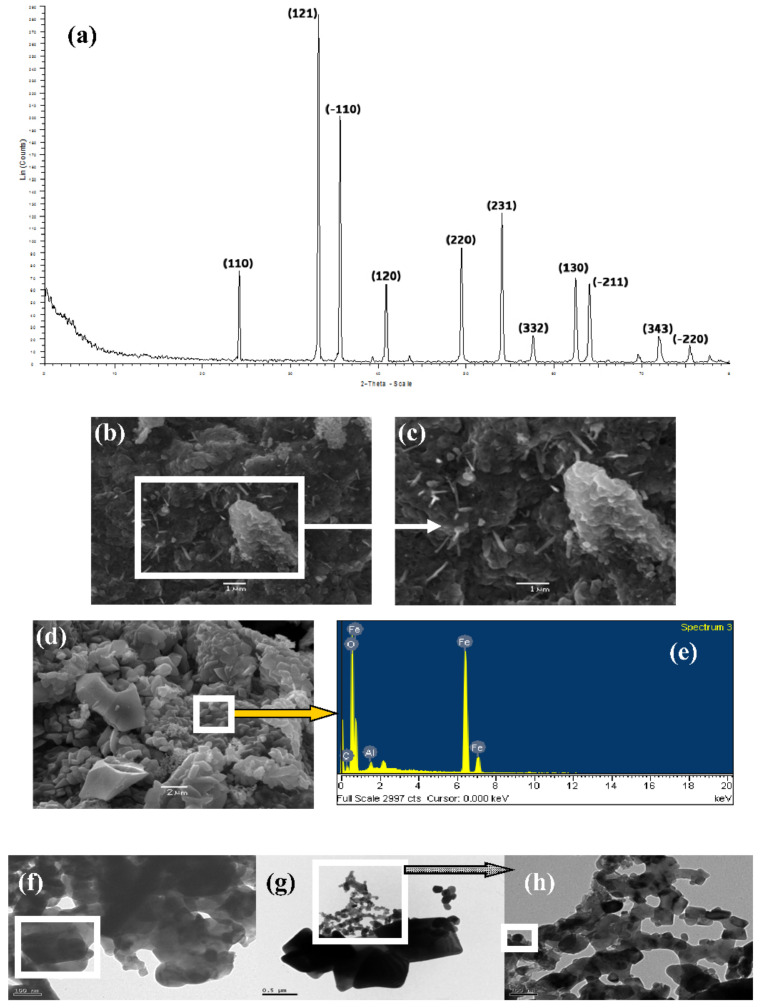
XRD (**a**), SEM analysis (**b**–**d**), EDS (**e**), and TEM image (**f**–**h**) for the pyrolytic product from Chitosan●(FeCl_3_)_n_ 1:1, hematite-Fe_2_O_3_.

Among the different iron oxides, one of the most common is Fe_2_O_3_ hematite [78,79]. For the study with Fe, the following macromolecular complexes were prepared: Chitosan·(FeCl_2_)_n_, chitosan·(FeCl_3_)_n_, PS-co-4-PVP·(FeCl_2_)_n_ and PS-co-4-PVP·(FeCl_3_)_n_ with molar ratios 1:1, 1:5, and 1:10. A complete study was performed using the following parameters: nature of the polymer, oxidation state of the iron salts, and the metal/polymer ratio. In all the cases, Fe_2_O_3_ (hematite) was obtained. A representative XRD for the pyrolytic product from chitosan·(FeCl_3_)_n_ 1:1, their SEM and TEM images are shown in Figure 6. XRD data were indexed according to the hematite structure [78,79,80,81,82,83]. SEM images (Figure 6b–d) exhibit varied irregular shapes, typical of solid-state methods, but show the correct Fe and O content atoms as shown in the EDS analysis (Figure 6e). Consistently, their TEM analysis exhibits agglomerates containing the smallest nanostructures joined in linear dispositions (Figure 6f–h). Chitosan with the FeCl_2_ salt induces the smallest Fe_2_O_3_ nanoparticle size, while for the PS-PVP polymer, the smallest nanoparticles were induced with FeCl_3_. The 1:1 molar ratio precursor also exhibits the smallest nanoparticles for both polymers.

##### Cobalt

**Figure 7:**
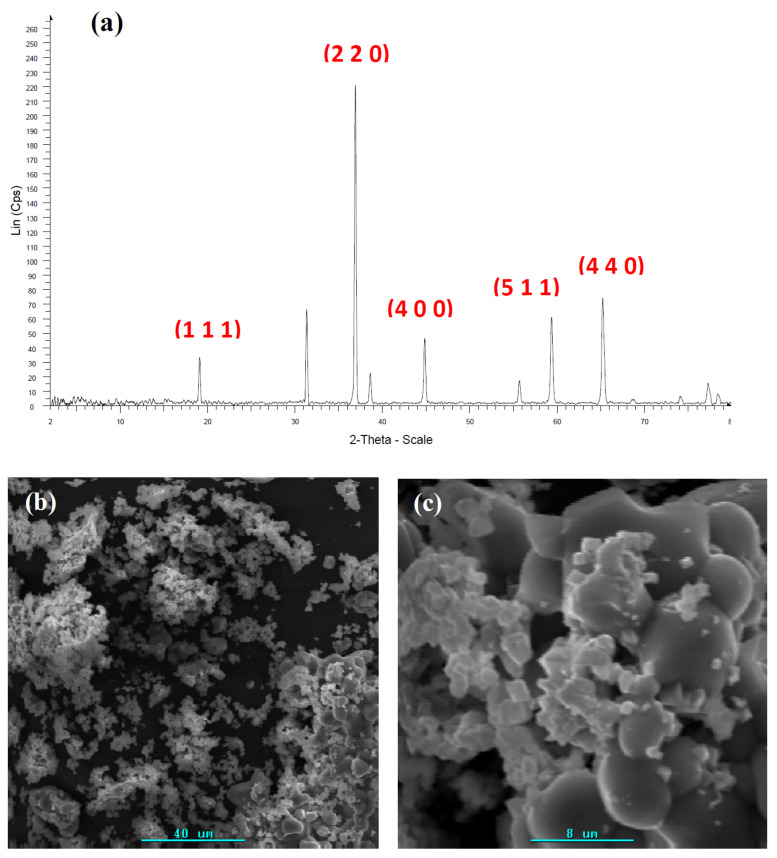
XRD pattern (**a**) and SEM images (**b**,**c**) for the pyrolytic product from Chitosan·(CoCl_2_)_n_.

Among the different cobalt oxides, Co_3_O_4_ and CoO [84,85,86,87,88,89] are the most common, being Co_3_O_4_, for example, used as a supercapacitor electrode material [86]. In this case, chitosan·(CoCl_2_)_n_ and PS-co-4-PVP·(CoCl_2_)_n_ precursors were selected. XRD analysis shows the presence of Co_3_O_4_ pure phase [84,85], as shown in Figure 7a. SEM images indicate after pyrolysis a dense morphology composed of joined grains to form a 3D arrangement. A similar thermal synthetic approach has been reported [87,88,89]. As normally found in solid-state methods, morphology with different and varied shapes and sizes are observed. In addition, a solid-state route starting from Co(II) salts has also been reported [84].

##### Nickel

**Figure 8:**
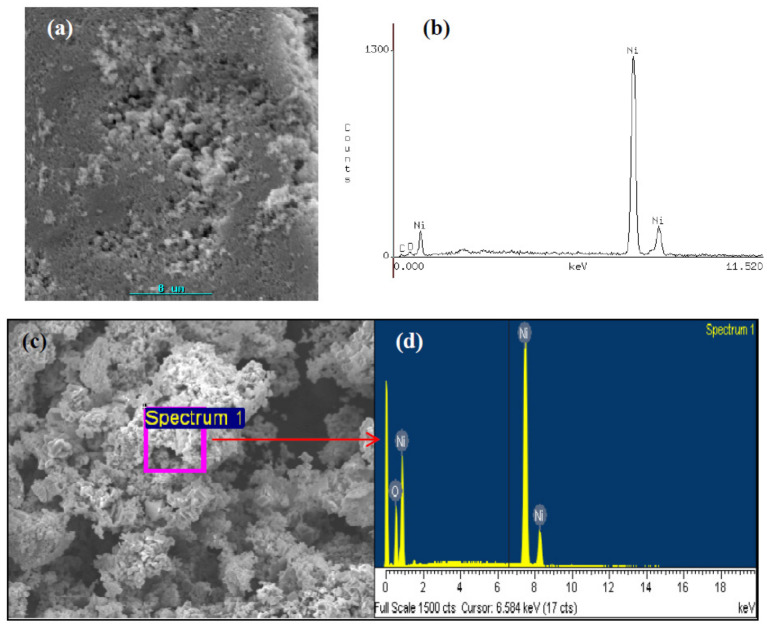
SEM image (**a**) and EDS analysis (**b**) from the pyrolytic product from 1:1 Chitosan·(NiCl_2_)_n_ precursor (**b**) and SEM image (**c**) and EDS analysis from (**d**) the 1:1 PS-co-4-PVP·(NiCl_2_)_n_ precursor.

For this study, we have selected the chitosan·(NiCl_2_)_n_ and PS-co-4-PVP·(NiCl_2_)_n_ precursors. XRD analysis clearly shows the presence of NiO pure phase. SEM image exhibits a porous morphology, as shown in Figure 8a. EDS analysis corroborates the presence of Ni and O. For the PS-co-4-PVP·(NiCl_2_)_n_ precursor, a more porous morphology was observed (Figure 8c). Their EDS analysis also indicates the presence of Ni and O, see Figure 8d.

NiO is a p-type semiconductor with a band-gap in the range 3.6–4 eV [60]. For their preparation, several solution methods [60] have been reported. In addition, a solid-state thermal method starting from [Ni(en)_3_][NO_3_]_2_ [29] has been described. The method reported here allowed us to prepare NiO from inexpensive and commercially available polymer and Ni salts. Using chitosan·(NiCl_2_·H_2_O)_x_ and PS-co-4-PVP·(NiCl_2_·6H_2_O)_x_ precursors [65] and by thermal treatment at 800 °C under air, pure NiO phase was obtained. The band-gap of the as-prepared NiO is 4.15 eV for the one obtained from both Chitosan and PS-co-4-PVP precursors [60]. For the semiconductor metal oxides, their band-gap value dictates their photocatalytic activity [89,90,91,92]. In fact, degradation of methylene blue was 71% and 68% using NiO obtained from Chitosan and PS-co-4-PVP precursors [60].

#### 2.3.2. Noble and Precious Metals

Noble metals such as Au, Pt, and Ag and metal oxides of the precious metal such as Ir, Rh, and Re can also be obtained using the same method. All these metal and metallic nanoparticles can be obtained by pyrolysis of the respective macromolecular chitosan·(ML_x_) and PS-co-4-PVP·(ML_x_) precursors using the appropriate metallic salts ML_x_ = AuCl, AuCl_3_, AuC_6_F_5_, Au(PPh_3_)Cl for Au, Ag(CF_3_SO_3_)_2_ for Ag, PtCl_2_ for Pt, IrCl_3_ for Ir, RhCl_3_ for Rh and ReCl_3_ for Re.

##### Gold

**Figure 9:**
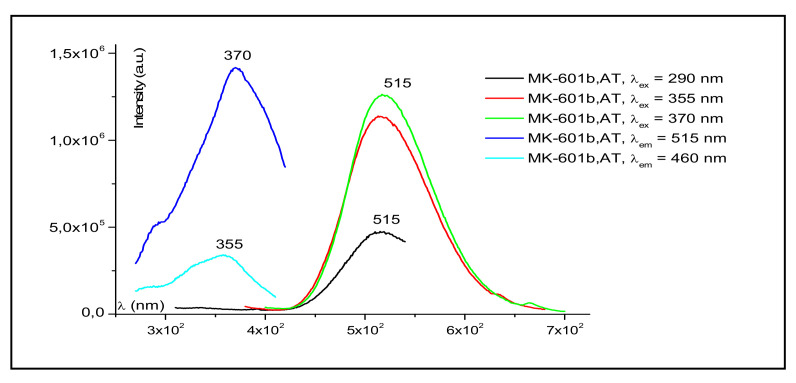
Luminescence spectrum of PS-4-co-PVP·AuC_6_F_5_ at several excitation wavelengths.

The facile reaction of the Au metallic salts = AuCl, AuCl_3_, AuC_6_F_5_, Au(PPh_3_)Cl with the PS-4-co-PVP polymer leads to the luminescent macromolecular complexes PS-4-co-PVP·AuCl_3_, PS-4-co-PVP·AuCl, PS-4-co-PVP·AuC_6_F_5_, and PS-4-co-PVP·Au(PPh_3_)Cl. The observed luminescence for PS-4-co-PVP·AuC_6_F_5_ with a maximum around 550 nm, as shown in Figure 9, arises probably from the Au(I)-Au(I) interactions [93]. Interestingly, the macromolecular complex PS-4-co-PVP●(AuCl_3_)_n_ does not present this luminescent behavior.

**Figure 10:**
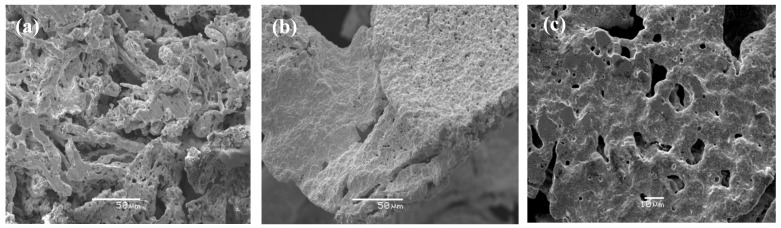
SEM images for the different (AuX_n_)_n_ samples: (**a**) AuCl, (**b**) AuCl_3_, and (**c**) Au(C_6_F_5_).

The pyrolysis of all these macromolecular precursors affords metallic Au° foams with pore size depending on the nature of the Au salt joined to the polymer chain [94] (Figure 10).

Metallic sponges (or foams) of macroporous metals have attracted great attention due to their unusual and peculiar properties such as mechanical strength and stiffness [95]. These materials can be prepared by several solution methods as dealloying of M/M′ alloys and by forming a metal organic composite and eliminating the organic part by dissolution or calcination. However, no solid-state approximations have been reported. The method described here could be a novel and reliable way to prepare metallic foams from different noble metals.

##### Silver

Several solution methods for preparing Ag° nanoparticles have been reported but relatively scarce from solid-state methods [96,97,98]. For Ag° containing precursors as Ag(CF_3_SO_3_)·Chitosan, Ag was obtained as shown in the XRD pattern [94]. Their morphology indicates a foam-like shape. Finally, EDS analysis confirmed the presence of Ag as a pure single phase.

##### Platinum

Nanostructured Pt nanoparticles are very important, for example, in the catalysis of fuel cells, sensors, and the petroleum and automotive industries due to their high catalytic activity and stability [98].

**Figure 11:**
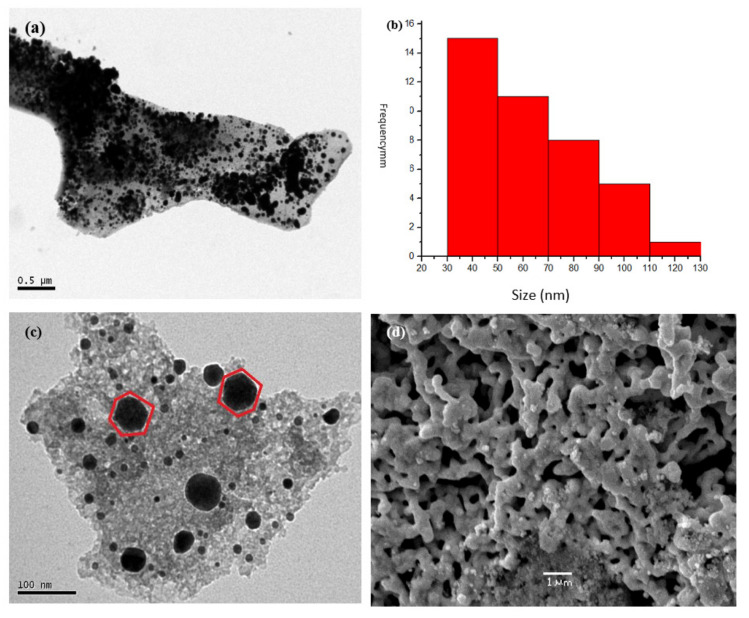
TEM image (**a**), particle size histogram, (**b**), TEM image in a magnified area (**c**), and an SEM image (**d**) for the pyrolytic products from the PS-co-4-PVP·(PtCl_2_)_n_ precursors. Adapted from reference [99].

Pt nanoparticles were obtained from the chitosan·(PtCl_2_)_n_ and PS-co-4-PVP·(PtCl_2_)_n_ precursors [99]. As shown in Figure 11, the obtained Pt nanoparticles exhibit varied shapes and sizes, some of them with the typical truncated octahedron [99,100]. This also can be viewed as an image of a cubohedral structure viewed along the [101] zone axis.

The smallest particle size (6 nm) was obtained from the pyrolytic product from the chitosan·(PtCl_2_)_n_ precursor in a 1:1 molar ratio. In spite of numerous preparation methods of Pt nanoparticles reported [101,102,103], few solid-state methods have appeared.

Another interesting aspect is the “foam-like” morphology observed for pyrolytic products from the precursors 1:5 ratio PS-co-4-PVP·(PtCl_2_)_n_. Few metallic Pt sponges materials have been reported, being this the only solid-state route for this type of material [95].

##### Iridium

Among the metals of the periodic table, the precious as Ir is one of the most catalytically active [104]. Their activity is hugely enhanced at the nano level [105,106,107]. This metal, as well as its metal oxides, exhibits a high catalytic activity [104,106]. Although isolated solution preparation methods for nanostructured Ir oxides are well documented [108,109,110,111,112,113,114], no solid-state general methods to prepare IrO_2_ nanostructured have been shown. IrO_2_ is generally prepared from an Ir salt. The relative fraction of IrO_2_/Ir depends on the temperature, producing Ir_2_O_3_ at temperatures between 250 and 400 °C, and then obtaining pure IrO_2_ at temperatures above 600 °C [108,109,110,111,112,113,114]. Using our method, we have obtained a unique nanostructured phase of IrO_2_ [63]. The IrO_2_ nanoparticles were prepared by thermal treatment of the macromolecular chitosan·(IrCl_3_)_X_ and PSP-4-PVP·(IrCl_3_)_X_ precursors. The nature of the polymeric precursor is acting as a solid-state template and influences the size of the iridium dioxide but not significantly the morphology, and the obtained IrO_2_ nanoparticles are about 15 nm.

##### Rhodium

Rhodium is another catalytically active precious metal [115]. Their activity is also hugely enhanced at the nano level [115,116]. Among these various noble metals, rhodium plays an important role in various catalytic applications [117,118]. However, the catalytic mechanism of rhodium-containing materials is still elusive. Recent investigations suggest that the active centers could be rhodium oxide rather than rhodium [118,119,120,121,122]. The most common rhodium oxides are Rh_2_O_3_ and RhO_2_. Although these rhodium oxides have a wide range of applications in catalysis, scarce preparation methods of nanostructured Rh_2_O_3_ and RhO_2_ have been reported, and their morphological and size control is vaguely known [7,8,9,118,119,120,121,122].

A nanostructured Rh/RhO_2_ mixture was easily obtained by thermal treatment of the macromolecular chitosan·(RhCl_3_)_X_ precursor, while the pure Rh_2_O_3_ was obtained from pyrolysis of the PSP-4-PVP·(RhCl_3_)_X_ precursor [64]. The nature of the polymeric precursor acting as a solid-state template does not significantly influence the morphology of the Rh and their metal oxide. The average size of the as-obtained products is in the range of 20 nm.

#### 2.3.3. Representative Metals

SnO_2_ and ZnO are two of the most used materials in sensors [123,124,125]. SnO_2_ is a wide band-gap n-type semiconductor with great importance in several technological applications such as gas sensing, Li-ion batteries, and solar cells [124]. As a sensor, its main application is in H_2_ and CO detection. In addition, nanostructured ZnO is one of the most promising nanomaterials for sensors due to its biocompatibility, chemical and photochemical stability, high specific surface area, optical transparency, electrochemical activity and high electron mobility. ZnO has been employed for the detection of biological molecules [126].

SnO_2_ and ZnO were prepared by pyrolysis of macromolecular complexes: PS-co-4-PVP·(SnCl_2_)_n_ and PS-co-4-PVP·(ZnCl_2_)_n_ in several molar ratios under air at 800 °C [53]. For ZnO agglomerates, the respective hexagonal and cubic structures were observed with typical sizes of 30–50 nm for a precursor mixture ratio of 1:1.

**Figure 12:**
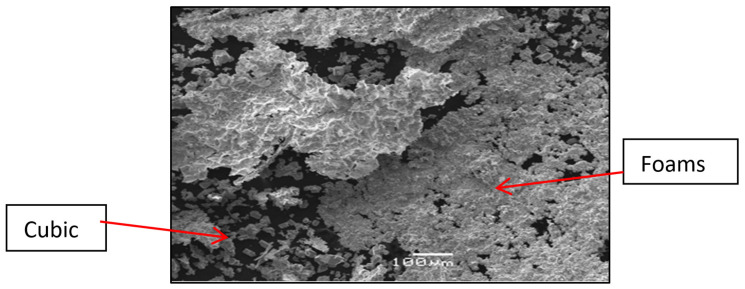
SEM image for the pyrolytic precursor from PS-co-4-PVP·(ZnCl_2_)_n_ in ratio 1:5. Adapted from reference [53].

Figure 12 shows, for instance, the SEM image for the pyrolytic precursor from PS-co-4-PV·(ZnCl_2_)_n_ in 1:5 ratio where zones with “metallic foams” as well as cubic morphologies were observed.

**Figure 13:**
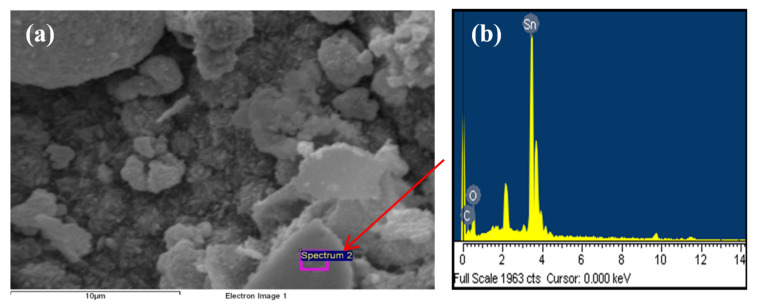
SEM image (**a**) and EDS analysis (**b**) of the SnO_2_ obtained from PS-co-4-PVP·(SnCl_2_)_n_ precursor.

For the SnO_2_ semiconductor, the oxide was prepared from the PS-co-4-PVP·(SnCl_2_)_n_ precursor. The nanostructured SnO_2_ exhibits morphologies and particle sizes depending on the molar ratio of the SnCl_2_:PS-co-4-PVP. When a larger weight fraction of the inorganic salt is used in the precursor (1:1), larger crystalline crystals are found for each oxide. As shown in Figure 13, the SEM image indicates an irregular morphology, including some “foam” shapes. EDS analysis confirmed the presence of Sn and O atoms.

Nanostructured ZnO was also obtained from the macromolecular chitosan·(ZnCl_2_) and PSP-co-4-PVP·(SnCl_2_)_n_ complexes [29].

#### 2.3.4. Rare Metals

Rare earth compounds have drawn attention due to their unique properties and promising application in, for example, UV-shielding, luminescent display, optical communications, biochemical probes, and medical diagnostic [127,128,129,130]. Among the rare earth compounds, metal oxides showed a variety of attractive features for applications in several fields of technology. However, few general preparation methods have been reported [131]. Using the above-described method, we can obtain rare earth oxide nanostructured compounds, although in some cases, the lanthanum oxyhalides were also obtained instead of the expected oxide Ln_2_O_3_. LnOX (Ln = lanthanide element, X = halide) compounds have unique and excellent characteristics in electrical, magnetic, optical and luminescent properties [132,133].

**Figure 14:**
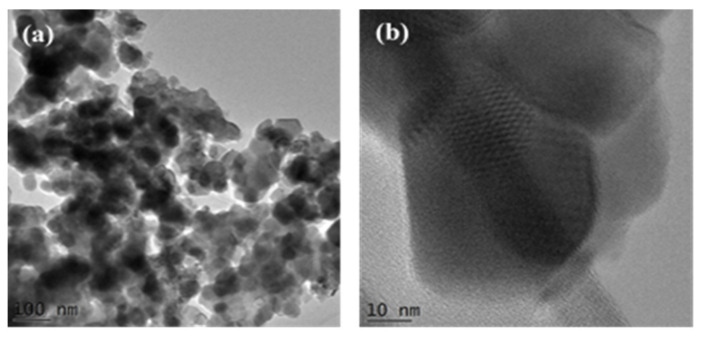
TEM image (**a**) and HRTEM (**b**) image of pyrolytic product from the precursor PSP-co-4-PVP·(Ce(NO_3_)_3_)_n_.

Pyrolysis products of the macromolecular PSP-co-4-PV·(ML_n_)_n_ and chitosan●(ML_n_)_n_ (M = lanthanide metal) complexes afford products of composition M_2_O_3_ or MOCl depending on the nature of the ML_n_ salt. When ML_n_ is MCl_3_, the product is the oxychloride MOCl, while when ML_n_ is M(NO_3_)_3_ or M_2_(SO_4_)_2_, the respective M_2_O_3_ is obtained. For instance, pyrolysis of the macromolecular PSP-co-4-PVP·(Ce(NO_3_)_3_)_n_ precursor gives rise to CeO_2_. The different obtained materials were identified by XRD. TEM shows the typical arrangements of nanoparticles somewhat agglomerated, as is displayed in Figure 14a. HRTEM images in Figure 14b confirm that the method allowed agglomerates of single-crystal nanoparticles of CeO_2_.

**Figure 15:**
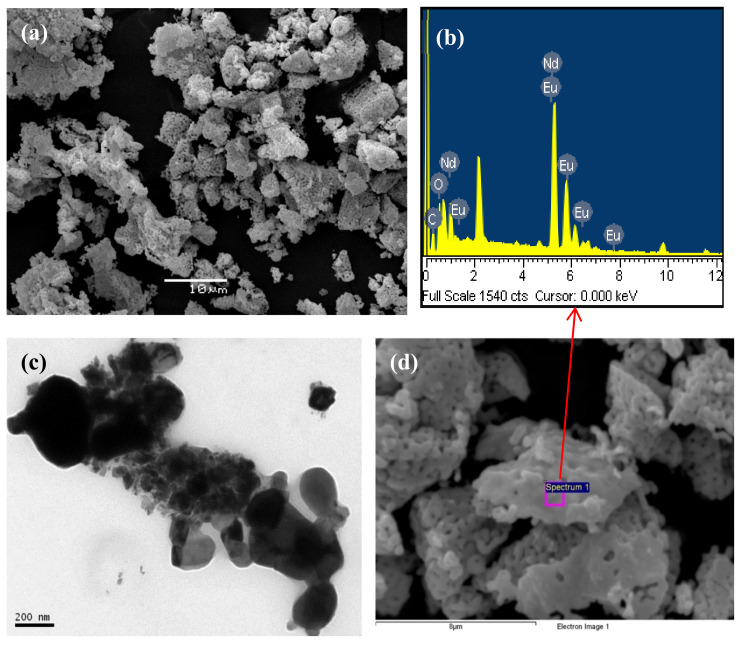
SEM (**a**,**d**), EDS (**b**), and TEM image (**c**) of the pyrolytic products from Eu^3+^ doped, Chitosan·NdCl_3_.

On the other hand, pyrolysis of chitosan●NdCl_3_ and PSP-co-4-PVP·NdCl_3_ give rise to NdOCl in both cases, as confirmed by XRD [60]. In some cases, a mixture with Nd_2_O_3_ oxide was also observed. SEM image exhibits a 3-D metallic foam morphology for the pyrolytic products from chitosan·NdCl_3_. EDS confirmed the presence of Nd, O, and Cl in agreement with the proposed formula. TEM image displayed irregular shapes with varied sizes [60], as shown in Figure 15c.

It is remarkable that Eu^+3^ doped NdOCl/Eu_2_O_3_ materials obtained from pyrolysis of chitosan●NdCl_3_/EuCl_3_ exhibit a 3D metallic foam structure (Figure 15) [57]. Interestingly, we observed the presence of Eu, in addition to the expected Nd, O, and Cl atoms, in the respective EDS (Figure 15b). The solid-state luminescence of this system shows the main emission line around 566 nm assigned to the ^5^D_0_ → ^2^F_2_ transition [57].

#### 2.3.5. Actinides

Among the different actinide oxides, thoria is an important and promising material used as a ceramic catalyst sensor in solid electrolytes, catalysis, and optical materials, as well as in the traditional nuclear industry [134,135,136,137,138]. In spite of this, few papers related to the preparation and properties of nanostructured ThO_2_ have appeared. We have prepared nanostructured ThO_2_ from the chitosan·Th(NO_3_)_4_ and PS-co-4-PVP·Th(NO_3_)_4_ precursors [66]. The morphology and the average size of the as-obtained ThO_2_ depend on the Chitosan and PS-co-4-PVP polymer forming the precursor. A total of 50 and 40 nm average sizes were observed from the Chitosan and PS-co-4-PVP polymer precursors. The as-obtained thoria exhibits the expected luminescence with a dependence on their intensity emission maxima and the nature of the precursor polymer.

## 3. Incorporation of Metallic and Metal Oxides into Solid Matrix

Various practical applications, for instance, catalysis, involving solid-state devices are formed by nanoparticles and/or nanostructured inside a solid matrix as SiO_2_, TiO_2_, Al_2_O_3_, glasses, and so on [139,140]. We have designed a solid-state methodology to prepare different composites: M/M′_x_O′_y_ and M_x_O_y_/M′_x_O′_y_ with M′_x_O′_y_ solid matrix, i.e., SiO_2_, TiO_2_, Al_2_O_3_, under air thermal treatment of the chitosan·MLn//M′_x_O′_y_ and PS-co-4-PVP·MLn//M′_x_O′ precursors. Using this synthetic methodology, we have been able to prepare several metallic nanoparticles as well as metal oxides included in solid matrices.

As known in catalysis, the inclusion of M°, as well as MxOy inside solid matrices, induces stability of the catalytic material as well as a greater surface area of the catalyst within the solid matrix [139,140]. Au° and the bimetallic Au°/Ag° were incorporated in silica to give Au°//SiO_2_ [94] and Au°/Ag°//SiO_2_ [56].

**Figure 16:**
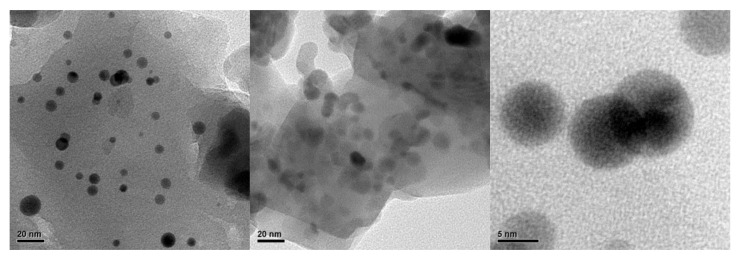
HRTEM images of Au nanoparticles inside SiO_2_ from (PS-co-4-PVP)•(AuCl_3_)_n_•(SiO_2_)_n_ precursor. Adapted from reference [94].

**Figure 17:**
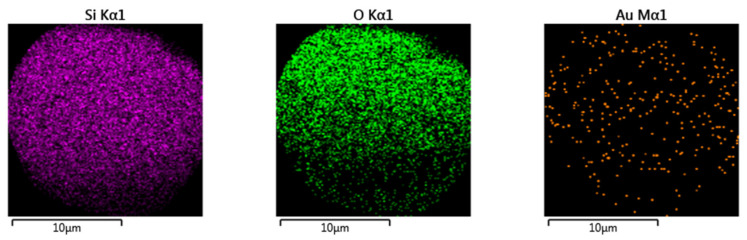
EDS mapping by elements of the Au/SiO_2_ nanocomposite from the precursor Chitosan•(AuCl_3_)_n_·(SiO_2_)_n_ in 1:1 molar ratio polymer/metal. Adapted from reference [94].

Nanoparticles of about 5 nm can be observed in Figure 16. The distribution of the Au° nanoparticles inside silica was examined by SEM-EDS mapping, as seen in Figure 17.

**Figure 18:**
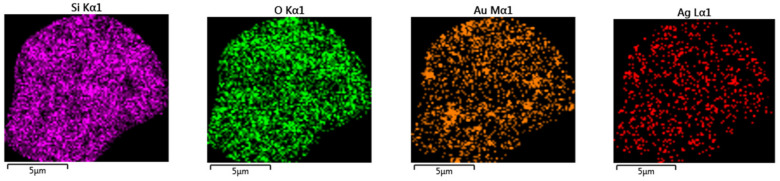
EDS mapping for the Au/Ag//SiO_2_ composite from the macromolecular Chitosan·(AuCl_3_/AgSO_3_CF_3_)_n_·SiO_2_ precursor 1:1. Adapted from reference [94].

From this figure, regular distribution of the Au° nanoparticles inside silica was observed. In addition, the inclusion of the bimetallic Au°/Ag° nanoparticles was performed by pyrolysis under air at 800 °C of the PSP-4-PVP•(AuCl_3_/AgSO_3_CF_3_)_n_·SiO_2_ and chitosan·(AuCl_3_/AgSO_3_CF_3_)_n_·SiO_2_ precursors. The relative distribution of the Au/Ag bimetallic nanoparticles inside silica was observed by the EDS mapping shown in Figure 18.

The inclusion of the Ag° nanoparticles inside SiO_2_ was also made by pyrolysis of the respective (PS-co-4-PVP)·(AgNO_3_)_n_•(SiO_2_)_n_ and chitosan·(AgNO_3_)_n_•(SiO_2_)_n_ precursors [94]. Well dispersed Ag nanoparticles inside SiO_2_ were observed for the Ag/SiO_2_ composites obtained from chitosan•(AgNO_3_)n•(SiO_2_)_m_ and (PS-co-4-PVP)·(AgNO_3_)n•(SiO_2_)_m_, with particle sizes of 5 and 6 nm, respectively.

Furthermore, ZnO and SnO_2_ were also included in silica [58]. Pyrolysis of the ZnCl_2_·chitosan·SiO_2_ and SnCl_2_·chitosan·SiO_2_ precursors at 800 °C under air afford mixtures of Zn_2_SiO_4_ and SiO_2_, and pure SnO_2_, respectively. SnO_2_ nanoparticles are regularly distributed inside the silica matrix.

The inclusion of IrO_2_ into SiO_2_ was performed using a combined solution of the chitosan and PVP precursors using the sol-gel method [63]. Subsequent pyrolysis of the isolated solid-state chitosan·(IrCl_3_)_x_(SiO_2_)_y_ and PSP-4-PVP·(IrCl_3_)x(SiO_2_)_y_ give rise to the IrO_2_//SiO_2_ nanocomposites. The IrO_2_ particles are distributed uniformly inside the matrix of SiO_2_, leading to stable porous materials appropriate for high-temperature catalytic applications.

The same procedures were used for Re and Th. The inclusion of ReO_3_ into SiO_2_ was performed using a combined solution of the chitosan and PSP-4-PVP precursors by the sol-gel method [62]. Subsequent pyrolysis of the solid chitosan·(ReCl_3_)_X_(SiO_2_)_y_ and PSP-4-PVP·(ReCl_3_)_X_·(SiO_2_)_y_ precursors give rise to the ReO_3_//SiO_2_ nanocomposites. The as-obtained ReO_3_ nanoparticles inside SiO_2_ are as small as 1 nm. ReO_3_ nanoparticles are distributed uniformly inside the SiO_2_ matrix, leading to stable semiporous materials suitable for high-temperature catalytic applications. The inclusion of ThO_2_ inside SiO_2_ and TiO_2_ was achieved through a similar solid-state method [140,141,142]. The ThO_2_/SiO_2_ composites were prepared by pyrolysis at 800 °C under air of the chitosan●Th(NO_3_)_4_//SiO_2_ and PS-co-4-PVP·Th(NO_3_)_4_//SiO_2_ precursors [66]. On the other hand, ThO_2_/TiO_2_ composites were similarly prepared by pyrolysis at 800 °C chitosan·Th(NO_3_)_4_//TiO_2_ and PS-co-4-PVP·Th(NO_3_)_4_//TiO_2_ precursors. ThO_2_ particles exhibit a suitable dispersion inside the silica showing sizes of 250 nm and 950 nm depending on the chitosan or PS-co-4-PVP polymer precursors, respectively. SEM-EDS mapping analysis shows a regular dispersion of the thoria into the SiO_2_ and TiO_2_ matrices. The luminescent properties of the ThO_2_/SiO_2_ and ThO_2_/TiO_2_ composites show a dependence of their luminescence intensity, being the most intense with the TiO_2_ matrix.

On the other hand, the inclusion of NiO inside the SiO_2_, TiO_2_, Al_2_O_3_, Na_4.2_Ca_2.8_(Si_6_O_18_) matrices was also performed by solid-state under-air pyrolysis of the: Chitosan·(NiCl_2_·6H_2_O)_x/_/SiO_2_, PS-co-4-PVP·(NiCl_2_)_x/_/SiO_2_, chitosan·(NiCl_2_·6H_2_O)_x/_/TiO_2_, PS-co-4-PVP(NiCl_2_)_x/_/TiO_2_, chitosan(NiCl_2_·6H_2_O)_x/_/Al_2_O_3_, PS-co-4-PVP·(NiCl_2_)_x_/Al_2_O_3_, chitosan(NiCl_2_·6H_2_O)_x_//NiO/Na_4.2_Ca_2.8_(Si_6_O_18_) and PS-co-4-PVP(NiCl_2_)_x_//NiO/Na_4.2_Ca_2.8_(Si_6_O_18_) precursors [65]. The new composites were characterized by XRD, SEM/EDS, TEM, and HR-TEM. The size of the NiO nanoparticles obtained from the PSP-4-PVP precursors inside the different matrices follows the order of SiO_2_ > TiO_2_ > Al_2_O_3_. However, NiO nanoparticles obtained from the chitosan precursor do not present an effect on the particle size. It was found that the matrices (SiO_2_, TiO_2_, Al_2_O_3_, and Na_4.2_Ca_2.8_(Si_6_O_18_)) have a medium effect on the band-gap energy and also on the photocatalytic methylene blue degradation.

## 4. Photocatalytic Applications

Industrial plants generate increasing amounts of wastewaters, which often causes severe environmental problems [141,142]. Wastewaters produced in many industrial processes typically contain organic compounds that are toxic and not amenable to direct biological treatments [142]. There are huge numbers of different types of organic pollutants, including dyes, phenols, biphenyls pesticides, fertilizers, hydrocarbons, plasticizers, detergents, oils, greases, pharmaceuticals, proteins, carbohydrates, and so on [143]. Therefore, there is a great need to develop an efficient and cost-effective technique to reduce the concentration of organic pollutants before releasing the wastewaters into the aquatic environment. Currently, industrially available wastewaters treatment technologies such as adsorption and coagulation merely concentrate or separate these pollutants from water but do not completely “eliminate” or “destroy” them into biodegradable or less toxic organic compounds [144]. Other water treatments methods, such as chemical and membrane technologies, usually involve high operating costs and sometimes generate other toxic secondary pollutants [145]. Among the various physical, chemical, and biological technologies used in pollution control, including biological technologies, advanced oxidation processes such as photocatalysis are being increasingly adopted in the destruction of the organic contaminant due to their high efficiency, simplicity, suitable reproducibility, and ease of handling [146]. Heterogeneous photocatalysis possesses some critical advantages that have feasible applications in wastewater treatments, including:

(i) Ambient operating temperatures and pressure;
(ii) Complete mineralization of contaminants and their intermediates compounds without leaving secondary pollutants;
(iii) Low operating costs [146].

Among the most used photocatalyst are the nanostructured metal oxides [10,11]. However, their current preparation involves mainly in-solution methods [147,148,149,150,151,152,153,154], which present some problems in the isolation of the solid by elimination of the solvent as well as the elimination of the template and of the stabilizer [151].

Among the main applications of nanostructured metal oxides, the photocatalytic degradation of organic pollutants lies in the field of environmental remediation. The main characteristics that a suitable metal oxide photocatalytic system must include [145]:

1. An adequate band-gap;
2. Suitable morphology;
3. High surface area;
4. Stability and reusability.

**Figure 19:**
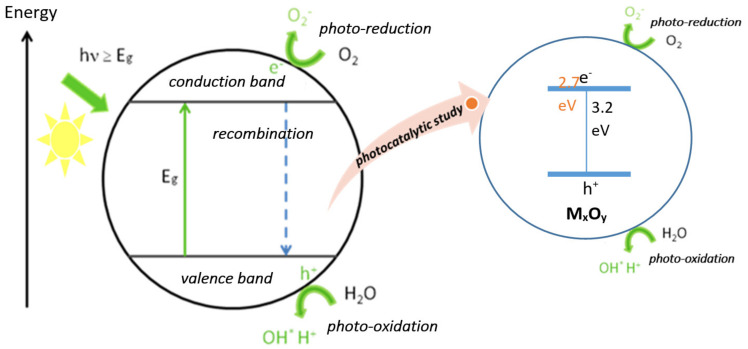
Photocatalytic activity of nanostructure metal oxides.

Semiconductor metal oxides having a band-gap near 3.2 eV are UV light active, while semiconductor metal oxides with a band-gap near 2.7 eV are visible light active [142]. Metal oxides exhibiting these features, such as vanadium, chromium, titanium, zinc, tin, and cerium, follow similar primary photocatalytic processes such as light absorption, which induce a charge separation process with the consequent formation of positive holes that are able to oxidize organic substrates. In this process, a metal oxide is activated by either UV light, visible light, or a combination of both, and photoexcited electrons are promoted from the valence band to the conduction bands, forming an electron/hole pair (e^−^/h^+^). The photogenerated pair (e^−^/h^+^) is able to reduce and/or oxidize a compound adsorbed on the photocatalyst surface. A schematic representation of these processes is shown in Figure 19.

The photocatalytic activity of metal oxides comes from two sources [148]:

1. Generation of •OH radicals by oxidation of OH^−^ anions;
2. Generation of O_2_^−^ radicals by reduction in O_2_.

Semiconductor nanostructured metal oxides have been widely used in photocatalytic redox processes because of their electronic configuration of the filled valence band (VB) and empty conduction band (CB). When exposed to a photon with energy exceeding the band-gap, hν > E_g_, it generates an electron-hole pair with one electron in VB pumped into CB, leaving a hole behind in VB. The generated holes in VB are of great oxidation capability, while the electrons in the CB have high reducing potential.

These highly reactive electrons and holes participate in photocatalytic organic degradation.

As mentioned above, the factors that are important for an efficient photocatalyst include an adequate band-gap, suitable morphology, high surface area, stability, and reusability. The achievement of these characteristics for a given nanostructured metal oxide will depend on its preparation method. For instance, TiO_2_ is one of the most used and efficient metal oxides for photocatalytic degradation of several organic dye pollutants [145]. However, its relative efficiency depends on the preparation method, which in turn determinates the band-gap, the morphology, the surface area, and their stability and reusability. In this context, our solid-state method could afford nanostructured metal oxides that can easily exhibit the above characteristics, yielding an efficient photocatalyst for the degradation of organic pollutants.

One of the advantages of the metal oxides obtained by the solid state described above is that they can be used directly in photocatalytic heterogeneous catalysis.

**Figure 20:**
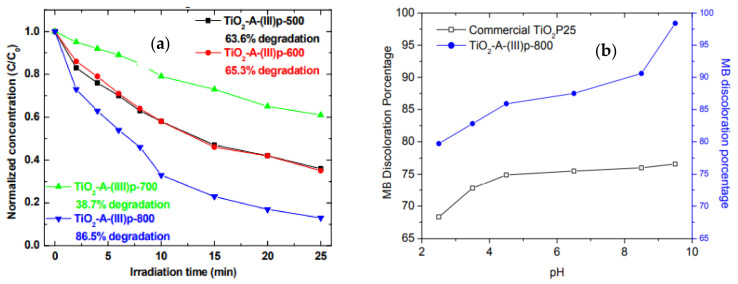
(**a**) Normalized concentration changing of MB as a function of time for all TiO_2_ photocatalyst obtained by precursors (Chitosan)•(TiOSO_4_) at different temperatures. (**b**) Effect of pH on MB (1 × 10^−5^ M) discoloration using our best TiO_2_.

For all TiO_2_ products described in Table 1, the most effective degradant of methylene blue was the anatase obtained from the precursor (chitosan)·(TiOSO_4_) at 800 °C [59]. This material achieved a 98% discoloration rate in only 25 min when the pH of the solution was 9.5, improving the efficiency of the standard photocatalyst Degussa P25 without the addition of other phases or dopants. Figure 20a shows the c/c_0_ vs. irradiation time for the TiO_2_ from (chitosan)·(TiOSO_4_) at several temperatures. At 800 °C, 86.5% discoloration rate in 25 min was observed. Optimization of the pH shows a 98% discoloration at pH 9.5, as shown in Figure 20b. From all different known TiO_2_ materials, the obtained using our solid-state method is one of the most efficient toward methylene blue degradation [59].

**Figure 21:**
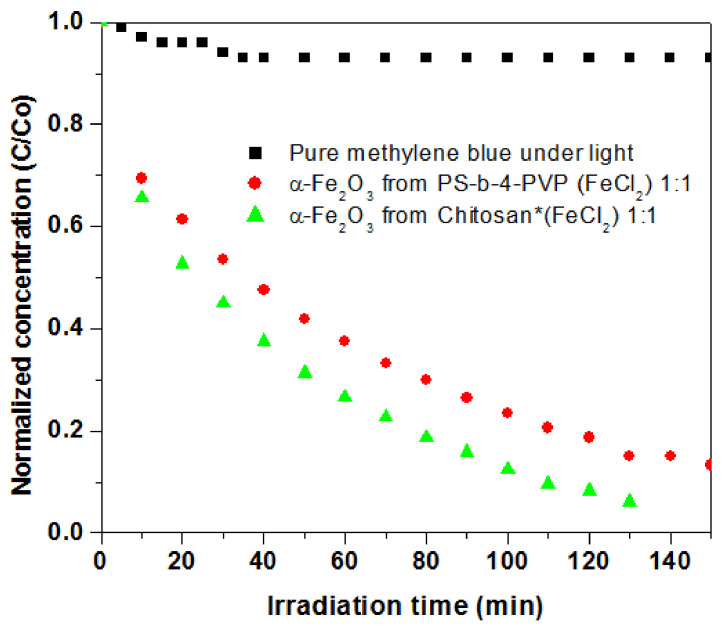
Normalized concentration changing of MB without catalyst, in the presence of a-Fe_2_O_3_·PS-co-4-PVP and in the presence of a-Fe_2_O_3_·chitosan. Adapted from reference [54].

Another photocatalytic-assayed system was hematite-Fe_2_O_3_ [54]. The nanoparticulate material obtained from chitosan·(FeCl_2_)_y_ 1:1 under the simulated sunlight (full visible spectrum) irradiation provides high rate degradation of MB by 73% in 60 min and >94% after 150 min, measured at 655 nm, as seen in Figure 21. The high photocatalytic efficiency can be due, in part, to the porous morphology of the hematite-Fe_2_O_3_ [54].

**Figure 22:**
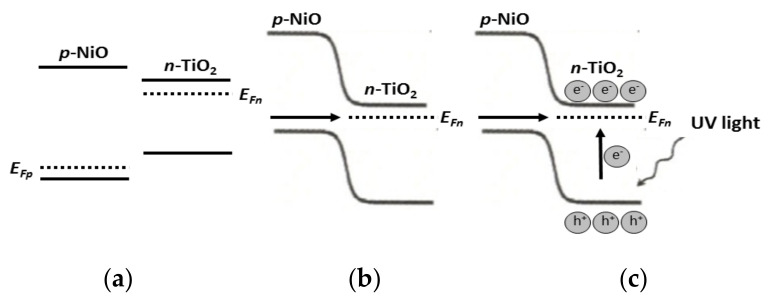
Schematic diagrams for (**a**) energy bands of p-NiO and TiO_2_ before contact, (**b**) formation of the p-n junction and its energy diagram at equilibrium, and (**c**) transfer of holes from n-TiO_2_ to p-NiO under UV irradiation.

On the other hand, NiO as well as their NiO/SiO_2_, NiO/TiO_2_, NiO/Al_2_O_3_ nanocomposites presenting band-gap values in the range 5.0–5.6 eV, see Table 2, predicts that they can be used as appropriate photocatalyst using UV irradiation [65]. In fact, NiO and their NiO/SiO_2_, NiO/TiO_2_, NiO/Al_2_O_3_ nanocomposites exhibit a satisfactory efficient photocatalytic behavior [65]. The higher methylene blue efficiency was for the NiO/TiO_2_ composite arising from the chitosan (NiCl_2_.6H_2_O)_x_//TiO_2_ precursor, see Table 3. This can be due to a p-n junction that can be formed acting NiO as p-NiO and TiO_2_ as n-TiO_2_. Therefore, it seems that the matrix is playing a crucial role for the NiO/TiO_2_ composite, and in this case, the NiO acts as the matrix rather than an active semiconductor, see Figure 22.

We have also studied the photocatalytic behavior of ReO_3_ prepared from the pyrolysis of the chitosan·(ReCl_3_)_X_ and PSP-4-PVP·(ReCl_3_)_X_ precursors [62]. The as-prepared ReO_3_ exhibits a moderated and high activity for ReO_3_ arising from chitosan and PSP-4-PVP precursors’ respectively. The composite ReO_3_//SiO_2_ prepared by solid-state pyrolysis of the chitosan·(ReCl_3_)_X_(SiO_2_)_y_ and PSP-4-PVP·(ReCl_3_)_X_(SiO_2_)_y_ precursors exhibit a moderate photocatalytic activity toward the degradation of methylene blue and similar to that of ReO_3_, see Table 4. This is the first report of the photocatalytic activity of ReO_3_ and ReO_3_//SiO_2_ composite.

We also tested the photocatalytic activity of IrO_2_ and their composite with SiO_2_ obtained by solid pyrolysis of the chitosan·(IrCl_3_)_X_, PSP-4-PVP·(IrCl_3_)_X_, chitosan·(IrCl_3_)_x_(SiO_2_)_y_, and PSP-4-PVP·(IrCl_3_)x(SiO_2_)_y_ precursors.

The oxide Rh_2_O_3_ and the mixture Rh/RhO_2_ have band-gap values of 3 and 3.7 eV, respectively, so they could have photocatalytic activity using UV irradiation [64]. In fact, Rh_2_O_3_ and the Rh/RhO_2_ mixture exhibit methylene blue degradation of 70% and 78% in 300 min, respectively. To the best of our knowledge, no photodegradation of pollutants using these type Rh oxides have been reported previously.

Finally, we have measured for the first time the catalytic activity of the thoria and of their ThO_2_/SiO_2_ and ThO_2_/TiO_2_ composites. As shown in Table 5, thoria prepared from the chitosan·Th(NO_3_)_4_ precursor exhibited an activity of 66% in 300 min while that thoria prepared from PS-co-4-PVP·Th(NO_3_)_4_ precursor presents activity of 67% degradation of MB in the same time. In addition, the photocatalytic efficiency of the ThO_2_/SiO_2_ and ThO_2_/TiO_2_ composites decrease significantly, as can be viewed from Table 5. The photocatalytic activity toward methylene blue degradation follows the order ThO_2_ > ThO_2_/TiO_2_ > ThO_2_/SiO_2_, which can be due to the decrease in the active sites of the surface as a consequence of the encapsulation of the ThO_2_ into TiO_2_. An additional reason could be the porous morphology of ThO_2_, which is encapsulated inside the SiO_2_ and TiO_2_ matrices [66].

## 5. Probable Formation Mechanism of Nanostructures Metallic and Metal Oxides

**Figure 23:**
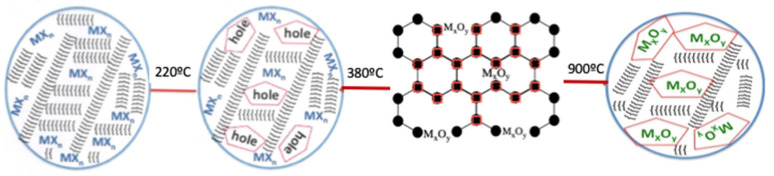
Schematic representation of the proposed mechanism of formation of the metal oxide nanoparticles. MX_n_ represents the general formula of the metallic salt coordinated to the Chitosan and PSP-4-PVP polymer, }}}}}}} represents the Chitosan and PSP-4-PVP polymer. M_x_O_y_ represents the respective metal oxides formed inside the graphite matrix. The given temperatures are referential general values.

Although the formation mechanism of nanoparticles in solution is well known [152,153,154], the studies of solid-state preparation methods are limited, and the parameters controlling both the size and the morphology of the formed nanoparticles are unknown. In this regard, we have evidenced that the pyrolysis of the {NP(OC_8_H_12_)_2_(OC_6_H_4_PPh_2_-Mn(CO)_2_(η^5^-C_5_H_4_Me)_2_} precursor occurs through the intermediate formation of a layered graphite host, which is formed in the first step of the thermal solid-state reaction [16]. In addition, the formation of the nanostructured Mn_2_O_3_ and Co_2_O_3_ compounds from their respective (chitosan)(MLn)_x_, MLn = MnCl_2_, and CoCl_2_ macromolecular complexes precursors was confirmed to occur through an intermediate state, a layered graphitic carbon matrix, which was observed by HRTEM and Raman measurements [55]. More recently, the formation of TiO_2_ using several pyrolysis temperatures was confirmed with the formation of a graphite intermediate [59]. Considering all studies, a general mechanism is proposed, as shown in Figure 23.

The first step on heating involves the formation of a 3D network [15] to produce a thermally stable matrix. This step is crucial because it offset the sublimation. In our system, the first heating step could involve a cross-linking of the Chitosan and PS-*co*-4-PVP polymers, giving a 3D matrix containing O-M-O and H_2_N-M-NH_2_ links (for the chitosan polymer) and (pyridine)N-M-N(pyridine) bonds for the PS-*co*-4-PVP polymer. The following steps involve the starting of the organic carbonization, producing holes where the nanoparticles begin to nucleate. According to TG/DSC analysis, this occurs at ~400 °C for the chitosan and 360 °C for PS-*co*-4-PVP polymer matrices. In this intermediate stage, a layered graphitic carbon host (detected in our previous work [16]) acts as a template where the nanoparticles grow. After complete combustion, this template disappears but always remains carbon residues appearing as an ultrathin carbon shell around the nanoparticles [16].

## 6. Concluding Remarks

Although there are several solid-state methods to prepare nanoparticles, scarce mechanism studies have been reported, being this is a pending challenge. In this sense, control of parameters such as size and morphology of the formed nanoparticles is not known. Using the proposed solid-state method from the chitosan·MXn and PS-co-4-PVP·MXn complexes as precursors offers a reliable, general, and easy way for obtaining metal and metal oxides from all the periodic table. Using the chitosan●MLn//M′_x_O′_y_ and PS-co-4-PVP●MLn//M′_x_O′ precursors, M/M′_x_O′_y_ and M_x_O_y_/M′_x_O′_y_ composites with M′_x_O_y_ solid matrices can be easily obtained. However, a most detailed study of the effect of the inclusion of the metal and metal oxides nanoparticles inside the M′_x_O_y_ matrix is still pending. In spite of that, several metal oxides obtained by the described solid-state method exhibit a satisfactory photocatalytic toward contaminant dyes as methylene blue. In any case, most of them need to be double-checked to obtain a general conclusion about the photocatalytic effectiveness of these oxides in order to improve them. It is also envisaged that the nanostructured metal oxides described here could significantly contribute to environmental decontamination.

## Figures and Tables

**Table 1:** Pyrolysis temperature, phase, particle size, and morphology for the TiO_2_ obtained from the different precursors at several temperatures.

**^a^ (Cp_2_TiCl_2_)** **·(Chiotosan)**				
**Temperature (°C)**	**Phase**	**Size (nm)**	**Dispersion (nm)**	**Morphology**
500	Anatase	11	+/−1	Lamellar
600	Mixture	13	+/−1	Lamellar
700	Mixture	11	+/−1	Porous mixture with sheets
800	Rutile	24	+/−2	Highly porous
**(Cp_2_TiCl_2_)** **·(PS-co-4-PVP)**				
**Temperature (°C)**	**Phase**	**Size (nm)**	**Dispersion**	**Morphology**
500	Anatase	13	+/−1	Porous and sheets
600	Anatase	25	+/−9	Granular
700	Anatase	32	+/−3	Porous and granular
800	Mixture	33	+/−2	Porous and sheet
**(TiOSO_4_)** **·(Chitosan)**				
**Temperature (°C)**	**Phase**	**Size (nm)**	**Dispersion**	**Morphology**
500	Anatase	27	+/−1	Nanoparticulated microfibers
600	Anatase	17	+/−5	Nanoparticulated microfibers
700	Anatase	32	+/−2	Nanoparticulated microfibers
800	Anatase	7 and 32	+/−1 and +/−2	Nanoparticulated microfibers
**(TiOSO_4_)** **·(PS-co-4-PVP)**				
**Temperature (°C)**	**Phase**	**Size (nm)**	**Dispersion**	**Morphology**
500	Anatase	11	+/−1	Irregular porous and microfibers
600	Anatase	20	+/−1	Irregular porous and microfibers
700	Mixture	35	+/−1	Nanoparticulated microfibers
800	Mixture	30	+/−1	Nanoparticulated microfibers
**(TiO(acac)_2_)** **·(Chitosan)**				
**Temperature (°C)**	**Phase**	**Size (nm)**	**Dispersion**	**Morphology**
500	Anatase	12	+/−1	Mainly porous
600	Anatase	14	+/−1	Irregular porous and sheets
700	Mixture	25	+/−1	Irregular porous and sheets
800	Mixture	18	+/−1	Mainly smooth
**(TiO(acac)_2_)** **·PS-co-4-PVP)**				
**Temperature (°C)**	**Phase**	**Size (nm)**	**Dispersion**	**Morphology**
500	Anatase	8	+/−1	Irregular porous
600	Mixture	11	+/−1	Irregular porous
700	Mixture	39	+/−3	Highly porous
800	Rutile	62	+/−2	Irregular porous and sheets

^a^ precursor formula.

**Table 2:** E_g_ values for NiO and NiO included in the SiO_2_, TiO_2_, Al_2_O_3_, and Na_4.2_Ca_2.8_(Si_6_O_18_) matrices Adapted from reference [65].

Composite	Precursor Formula	Eg(eV)
NiO	Chitosan·NiCl_2_	5.2
NiO	PSP-4-PVP·NiCl_2_	5.2
NiO/SiO_2_	Chitosan·NiCl_2_	5.0
NiO/SiO_2_	PSP-4-PVP·NiCl_2_	5.5
NiO/TiO_2_	Chitosan·NiCl_2_	5.2
NiO/TiO_2_	PSP-4-VP·NiCl_2_	5.2
NiO/Al_2_O_3_	Chitosan·NiCl_2_	5.4
NiO/Na_4.2_Ca_2.8_(Si_6_O_18_)	Chitosan·NiCl_2_	5.6

**Table 3:** Kinetic data for the photodegradation process of MB with NiO and NiO/SiO_2_, NiO/TiO_2_, NiO/Al_2_O_3_, and NiO/Na_42_Ca_2.8_(Si_6_O_18_) composites. Adapted from reference [65].

Photocatalyst	Discoloration Rate (%)
NiO-CHITOSAN	71%
NiO-PS-4-PVP	68%
NiO/SiO_2_-CHITOSAN	69%
NiO/SiO_2_-PS-4-PVP	48%
NiO/TiO_2_-CHITOSAN	91%
NiO/TiO_2_-PS-4-PVP	81%
NiO/Al_2_O_3_-CHITOSAN	45%
NiO/Na_4.2_Ca_2.8_(Si_6_O_18_)	75%

**Table 4:** Kinetic data for the photodegradation process of MB with ReO_3_ and ReO_3_/SiO_2_. Adapted from reference [62].

Photocatalyst	Photodegradation Rate Constant k (10^−3^ M·min^−1^)	Discoloration Rate (%)
ReO_3_-PS-4-PVP	2.8	64%
ReO_3_-Chitosan	2.8	53%
ReO_3_/SiO_2_-PS-4-PVP	3.7	67%
ReO_3_/SiO_2_-Chitosan	1.9	57%

**Table 5:** Photocatalytic efficiency of the different ThO_2_ composites.

Photocatalyst	Discoloration Rate (%)
ThO_2_-chitosan	67
ThO_2_-PS-4-PVP	66
ThO_2_/SiO_2_-chitosan	24
ThO_2_/SiO_2_-PS-4-PVP	25
ThO_2_/TiO_2_-chitosan	39
ThO_2_/TiO_2_-PS-4-PVP	27
